# Comparing and analyzing the accuracy and stability of calculating SF_6_ tracer gas mixing results in mine tunnels using RNG model and DES model through experiments

**DOI:** 10.1371/journal.pone.0314142

**Published:** 2024-12-23

**Authors:** Fang Xu, Jian Liu, Dong Wang

**Affiliations:** 1 College of Safety Science & Engineering, Liaoning Technical University, Huludao, Liaoning, China; 2 Key Laboratory of Mine Thermo-motive Disaster & Prevention, Ministry of Education, Huludao, Liaoning, China; 3 Liaoning Academy of Mineral Resources Development and Utilization Technical and Equipment Research Institute, Liaoning Technical University, Fuxin, China; Bina Nusantara University, INDONESIA

## Abstract

The tracer gas wind measurement method must measure the average concentration based on the known mixing distance of the tracer gas in order to accurately calculate the air volume. we established a T-shaped cross-ventilation duct with a square cross-sectional side of 0.3 m to simulate mine tunnels experimentally, and created the above duct model in Fluent software. Using the renormalization group (RNG k-ε) and detached eddy simulation (DES) models in Fluent to calculate the concentration and mixing distance of sulfur hexafluoride (SF_6_) tracer gas in air collection duct, and conducted SF_6_ blending experiments. In the first 7m stage of air collection duct, the SF_6_ mixing rate in the experiment was the fastest, while the RNG k-ε model was the slowest. However, after 7 meters, the mixing rate in the experiment rapidly slowed down, while there was no significant change in the RNG k-ε model. The rate of change of the DES model was between the above two results, but the concentration change was the most unstable. And the mixed distance results of the three were highly consistent. The results of this study provide a reference for selecting appropriate numerical simulation models for future calibration work of mine ventilation systems.

## 1 Introduction

Ventilation is key to ensuring the safety and health of underground production environments and personnel [1, 2]. Owing to their complex underground engineering, mines require proper ventilation to maintain a sustainable environment and avoid accidents. It is necessary to regularly measure the air volume of each main tunnel to ensure the normal operation of the mine ventilation system [3]. As production work in a mine progresses, the number and size of tunnels, and hence, the air volume within, change constantly, requiring the air dampers to be adjusted accordingly. For continued normal and orderly wind measurements once per day, when the ventilation system changes, the air volume of each tunnel must be first recalibrated [4]. At this point, no tunnel/ventilation parameters are calibrated, and hence, more accurate methods are needed for air volume measurement. Once calibration is completed, if the ventilation system remains unchanged in the future, a simpler wind measurement method with lower accuracy can be employed.

In the tracer gas wind measurement method, the tracer gas concentration is used to calculate the air volume with better accuracy compared to traditional wind measurement methods. This method does not require measuring the cross-sectional area and average wind speed of the tunnel, but only the stable release of a tracer gas in the tunnel [5]. Once the tracer gas diffuses evenly, its concentration is measured and the tunnel air volume is calculated according to the volume fraction law. This solves the problem of large errors due to the difficulty of measuring the tunnel cross-sectional area and the uneven wind speed distribution inside tunnels.

Sulfur hexafluoride (SF_6_) exhibits properties such as chemical stability, non-flammability, and non-toxicity. Moreover, its concentration can be accurately measured, making it an excellent tracer gas candidate [5, 6]. When using SF_6_ to test the overall mine air volume, multiple gas release points need to be arranged within the mine. For this purpose, the airflow entering the intake tunnel at an intersection must be mixed with the tracer gas.

As the SF_6_ concentration is measured at one or several points within the tunnel, uneven gas mixing SF_6_ can result in inaccurate average concentration, and hence, air volume measurements. The distance from the air inlet position of the tunnel to the point where SF_6_ and air are just evenly mixed (i.e., the mixing distance) is then determined. The area where air inlet distance ≥ mixing distance is the point where SF_6_ concentration becomes uniform. The SF_6_ concentration measured only at the mixing distance can be used to accurately calculate the air volume. Most intersecting tunnels in mines exist in the form of triple intersections [7, 8] and are divided into two forms, diverging and converging. In the converging form, the airflow from two tunnels converges into one tunnel. When measuring the airflow of the intersecting tunnels, one or no SF_6_ releasing device is needed to be installed in the inlet tunnel to calculate the combined airflow. This effectively reduces the SF_6_ cost and simplifies the airflow calculation.

Adel et al. used CO2 as a tracer to calculate the ventilation rate in the classroom, and conducted uncertainty analysis on the gas using three calculation methods: steady state, deck rate, and build up. They found that the uncertainty of steady state was the lowest [9]. Chen et al. discussed the tracer gas wind measurement method and proposed a formula for calculating the mixing distance [10]. Gao et al. studied the effects of tracer gas density and release rate on concentration distribution and ventilation effectiveness using computational fluid dynamics (CFD) method. When tracer gases with smaller release rates and density differences are released into stronger indoor airflow, their impact on indoor ventilation airflow is relatively small [11]. Widodo et al. studied the influence of the ratio of tunnel length and characteristic length on the SF_6_ concentration curve with time at a certain location in a tunnel [12]. Xu et al. established an experimental model of a mine tunnel and developed a proportional CFD model for the experimental model. We compared the concentrations of SF6 tracer gas at five points in the CFD model and experimental model, and proposed a method for detecting faults in mine ventilation systems using tracer gas [13, 14]. CFD method was used to optimize the release and sampling position of tracer gas, and the influence of release rate and time on the measured concentration was studied. And CFD methods can reduce the number of trial and error in engineering applications [15, 16]. However, most of these works focus on singular non-cross tunnels, and there is currently no research on the mixing distance of tracer gases in cross tunnels. When measuring cross tunnels, only SF6 needs to be released in one of the intake tunnels, and the air volume of the three tunnels can be calculated by knowing the release flow rate of SF6 and its mixing distance in the collection tunnel. This method can save SF6 release points and simplify the calculation process. When measuring the air volume of the entire mine air network, the study of the mixing distance of tracer gases in cross tunnels can simplify the calculation of air volume. Therefore, in this study, we compared the SF6 mixing process in cross tunnel models experimentally; obtained the mixing distance; and verified numerical simulation accuracy. The effect of wind speed and SF6 flow rate and amount at the release point on the mixing distance were also analyzed. In this T-shaped model, we constructed two intake ducts and one collection duct that intersect at a point, where one intake duct has a 90°angle with the collection duct, and the other intake duct has a 180° angle with the collection duct. The cross-section was a square with a side length of 0.3 m. Based on the gas lateral diffusion relation [10] and the Nikuradse experimental relation [17], a similarity relationship between the mixing distance, tunnel size, and wall roughness was derived. The measured mixing distance was compared to that computed by the numerical simulation of the mine tunnel to verify the accuracy. During practical applications of this mine wind measurement method, our numerical simulations and experimental results can provide data reference for the placement of SF6 concentration sensors, saving calculation time. We chose the renormalization group (RNG) k-ε and detached Eddy simulation (DES) models for this study. In the application of mine wind measurement and air volume calibration, the CFD method can calculate the mixing distance. In practical applications, the placement of concentration sensors can be roughly determined based on the CFD calculation results, saving the process and time of determining sensor positions on site, and also saving the use of tracer gases. This study has established an accurate, fast, and stable CFD model, providing a basis for determining the placement of sensors during air volume calibration.

## 2 Wind tunnel model numerical simulation setup

The method for calculating the air volume for tunnel intersections is detailed as follows: In one of intake tunnel, 100% volume fraction SF_6_ is continuously released at a constant flow rate using a release device and mixed uniformly with the air within. The SF_6_ concentration reaches the standard of mixes uniformly with the airflow at the air intake tunnel 2, and collection tunnel 3, concentration measurements are taken at the minimum and maximum concentration points at that location, and the average concentration is obtained by taking the average of the two maximum and minimum concentrations. Subsequently, based on the SF_6_ average concentration in the mine and in a single air intake tunnel, the tunnel air volume is calculated using the volume fraction law.

To visually compare with experimental data, an intersecting duct model of the same size and angles was constructed using the ICEM CFD software. The duct cross-section was set to a square with a side length of 0.3 m, with two intake air ducts measuring 2.5 m in length and a collection air duct measuring 40.0 m in length, used to simulate and calculate the SF_6_ mixing process and mixing distance. The grid adopted a highly accurate and fast hexahedral structure grid with a maximum grid edge length of 0.012 m, i.e., 1/25 times the cross-sectional edge length of the wind duct. We have conducted research on grid independence. When the maximum grid size is 0.012, at least 25 grids can be divided into the edges of the cross-section of the wind duct. When the maximum grid size is set to 0.0085, the edges of the cross-section can be divided into 36 parts. The concentration distribution and mixing distance calculated by the two grid sizes are almost indistinguishable. When the grid size has a minimal impact on the calculation results, a grid size of 0.012 was chosen to save computation time and space. Then determine the y+ (non-dimensional distance from the wall) of the grid close to the wall of the air duct. After verification, this grid size ensured both accurate calculation and fast operation. The grid near the duct wall needs to increase density, and the grid close to the duct wall should be in the log-law region, which is 30≤*y*+≤300 [18, 19]. The height of the first layer grid of the model was set to ensure 30 ≤ y+ ≤ 300 for wind speeds ranging from 6–12 m/s (the upper limit of the wind speed was set by the limitations of the fan and mixing blade speeds). The wall roughness was set to 0.5 mm, similar to the inner wall roughness of the experimental model. The Re for the model can be defined as [20]

$$Re=\frac{\mathrm{\rho vL}}{\mu },$$ (1)

where ρ is the gas density, kg/m^3^; v is the wind speed, m/s; L is the characteristic length, m; and μ is the gas dynamic viscosity, Pa·s.

The tunnel airflow is known to be in a completely turbulent state during actual mine ventilation. To similarly design the airflow state of the experimental air duct, we must introduce complete turbulence during the gas mixing experiments. In the experimental model, if Re ≥ 10^5^, a fully turbulent state can be achieved [21]. Using Eq (1), we found that this value for Re could be obtained by setting ν ≥ 5.159 m/s inside the experimental duct. The wind speed was fixed to a range of 6–12 m/s for the collection duct and the height of the first grid layer near the wall set to 0.012 ensuring 30 ≤ y+ ≤ 300, meeting the conditions for using the wall function [18].

The Navier Stokes (RNS) method is a low-cost approach, and the ke turbulence model also has the advantage of low computational cost in the RNS method. The RNG k-ε model takes into account the rotation and rotational flow conditions of the average flow based on the standard k-ε model, enabling the model to better predict the effects of transient flow and streamline bending. Additionally, it takes into account the low Reynolds number (Re) effects due to the wall roughness and gas viscosity in the near-wall region, improving accuracy for a wider range of flow and near-wall regions [22].

The transport equation of turbulent kinetic energy k in RNG k -ε model [23]:

$$\frac{\partial }{\partial t}(\mathrm{\rho k})+\frac{\partial }{\partial x_{i}}({\mathrm{\rho kv}}_{i})=\frac{\partial }{\partial x_{i}}(\alpha_{k}\mu_{\mathrm{eff}}\frac{\partial k}{\partial x_{j}})+G_{k}+G_{b}-\rho \varepsilon -Y_{M}+S_{k},$$ (2)

The transport equation of turbulent dissipation rate for RNG k-ε model:

$$\frac{\partial }{\partial t}(\rho \varepsilon )+\frac{\partial }{\partial x_{i}}({\rho \varepsilon \mu }_{i})=\frac{\partial }{\partial x_{i}}(\alpha_{\varepsilon }\mu_{\mathrm{eff}}\frac{\partial \varepsilon }{\partial x_{j}})+C_{\mathrm{1\varepsilon}}\frac{\varepsilon }{K}(G_{K}+C_{\mathrm{3\varepsilon}}G_{b})-C_{2\varepsilon }\rho \frac{\varepsilon^{2}}{k}-R_{\varepsilon }+S_{\varepsilon },$$ (3)

G_k_ represents the turbulent kinetic energy generated by the average velocity gradient, G_b_ represents the turbulent kinetic energy generated by buoyancy, and Y_M_ represents the contribution of wave expansion to the total dissipation rate in compressible turbulence.

u is velocity, m/s; ρ is density, kg/m3; α_k_ and α_ε_ are the reciprocal of the effective Prandtl numbers of k and e, respectively. C_1ε_ and C_2ε_ are model constants, where C_1ε_ = 1.42, C_2ε_ = 1.68.

The DES version of the Wray-Agarwal model is presented below [24].


$$\frac{\partial R}{\partial t}+\frac{\partial \mathrm{vR}}{\partial x_{j}}=\frac{\partial }{\partial x}[(\sigma_{R}R+\frac{\mu }{\rho })\frac{\partial R}{\partial x_{j}}]+C_{1}\mathrm{RS}+f_{1}C_{2\mathrm{k\omega}}\frac{R}{F_{\mathrm{DES}}^{2}S}\frac{\partial R}{\partial x_{j}}\frac{\partial S}{\partial x_{j}}-(1-f_{1})C_{2\mathrm{k\varepsilon}}\frac{R^{2}}{F_{\mathrm{DES}}^{2}S^{2}}{\left(\frac{\partial S}{\partial x_{j}}\right)}^{2},$$ (4)

Among them, F_DES_ is the characteristic length scale, and a new constant C_DES_ is introduced.


$$F_{\mathrm{DES}}=\max(\frac{l_{\mathrm{RA}\mathrm{NS}}}{l_{\mathrm{LES}}},1),$$ (5)

$$l_{\mathrm{RANS}}=\sqrt{\frac{R}{S}},$$ (6)

$$l_{\mathrm{LES}}=C_{\mathrm{DES}}\max(\Delta_{x},\Delta_{y}{,\Delta }_{z}),$$ (7)

**Fig 1:**
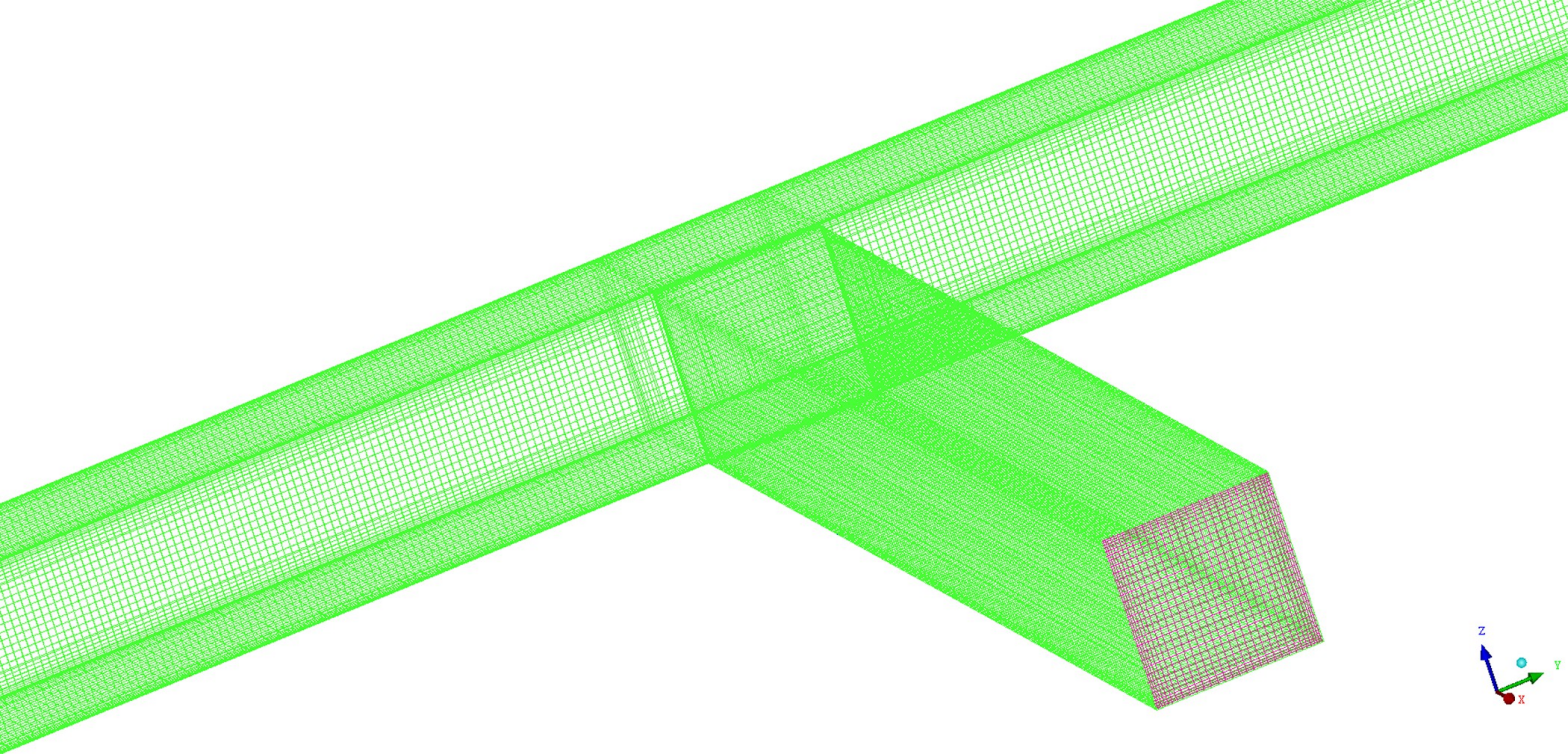
Duct structure and grid division.

**Fig 2:**
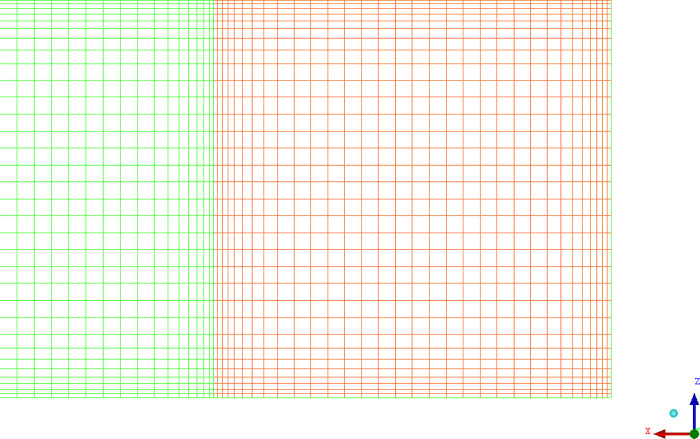
Grid division of air duct cross-section.

If eddy currents are considered in the calculation, the DES model can be used. The DES model adopts the large Eddy simulation (LES) algorithm in the turbulent core region, taking vortices into account, while the RANS (Reynolds-Averaged Navier-Stokes) algorithm was still used for the near-wall region [25]. Compared to the LES model, it could increase the grid size near the wall and reduce computational overhead. By comparing the results of these two algorithms with the experimental results, we analyzed their pros and cons and practical applicability. The constructed model and grid are shown in Figs 1 and 2.

We labeled the two intake air ducts as 1 and 2, and the collection air duct as 3, where duct 1 was parallel to duct 3 and duct 2 was perpendicular to duct 3.

At 25°C and standard atmospheric pressure, the densities of air and SF_6_ are 5.97 and 1.185 kg/m^3^, respectively [26], and the dynamic viscosities are 1.834 × 10^−5^ and 1.526 × 10^−6^ Pa·s [27].

The diffusion coefficient caused by molecular thermal motion is referred to here as the self-diffusion coefficient (*D*). The self-diffusion coefficient *D* of two different gases is calculated using FSG semi-empirical formulas [28]:

$$D=\frac{1.013\times 10^{-5}T^{1.75}{\left(\frac{1}{M_{A}}+\frac{1}{M_{B}}\right)}^{0.5}}{P{\left[{\left(\Sigma V_{A}\right)}^{\frac{1}{3}}+{\left(\Sigma V_{B}\right)}^{\frac{1}{3}}\right]}^{2}},$$ (8)

where *P* is the total air pressure (kPa), *T* is temperature (K), *M*_*A*_ and *M*_*B*_ are the molar masses of gases A and B (g/mol), respectively, and ∑*V*_*A*_ and ∑*V*_*B*_ are the diffusion volumes of gases A and B (cm^3^/mol), respectively.

The molar masses of air and SF_6_ are 28.959 and 146.06 g/mol, respectively, and previous studies have shown that the diffusion volumes of air and SF_6_ are 19.7 and 71.3 cm^3^/mol [29]. Therefore, when the temperature is 298.15 K and the pressure is 101.325 kPa, the diffusion coefficient of air–SF_6_ is approximately 9.281 × 10^−6^ m^2^/s. The parameter settings are shown in Table 1.

**Table 1:** CFD simulation parameter settings.

Parameter	Value
Temperature	298.15 K
Pressure	1 atm
Air Density	1.185 kg/m^3^
SF_6_ Density	5.97 kg/m^3^
Self-diffusion Coefficient	9.281×10^−6^ m^2^/s
Aerodynamic Viscosity	1.834×10^−5^ Pa·s
SF_6_ Dynamic Viscosity	1.526×10^−5^ Pa·s
Roughness Coefficient	0.5
Wall roughness	0.5 mm
Gravitational acceleration	9.8 m/s^2^
SF_6_ Concentration in duct 1	0 ppm
SF_6_ Concentration in duct 2	10 ppm
Wind Speed of Duct 1	3 m/s
Wind Speed of Duct 2	3 m/s

**Fig 3:**
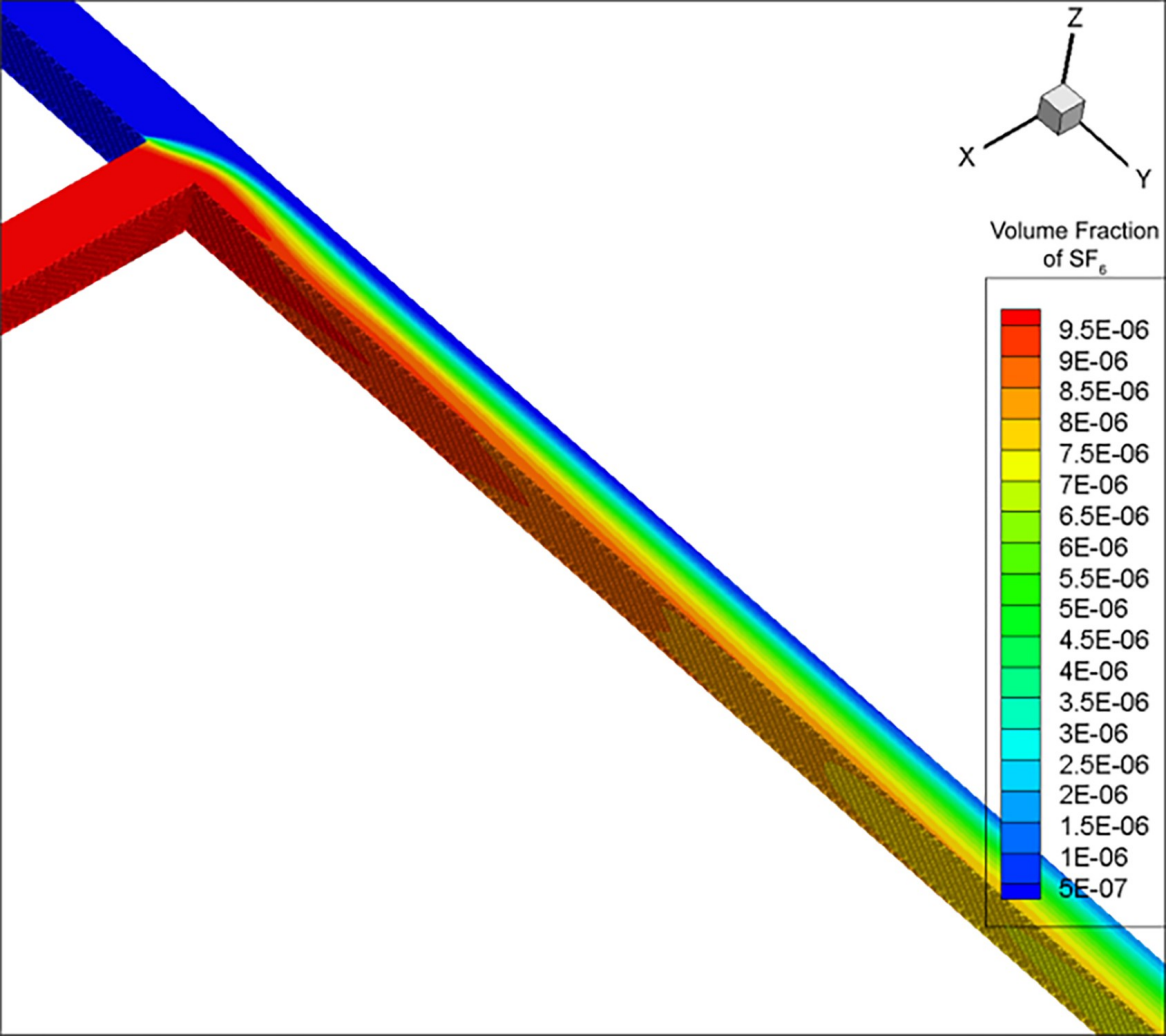
SF_6_ concentration distribution map calculated by the RNG k-ε model.

**Fig 4:**
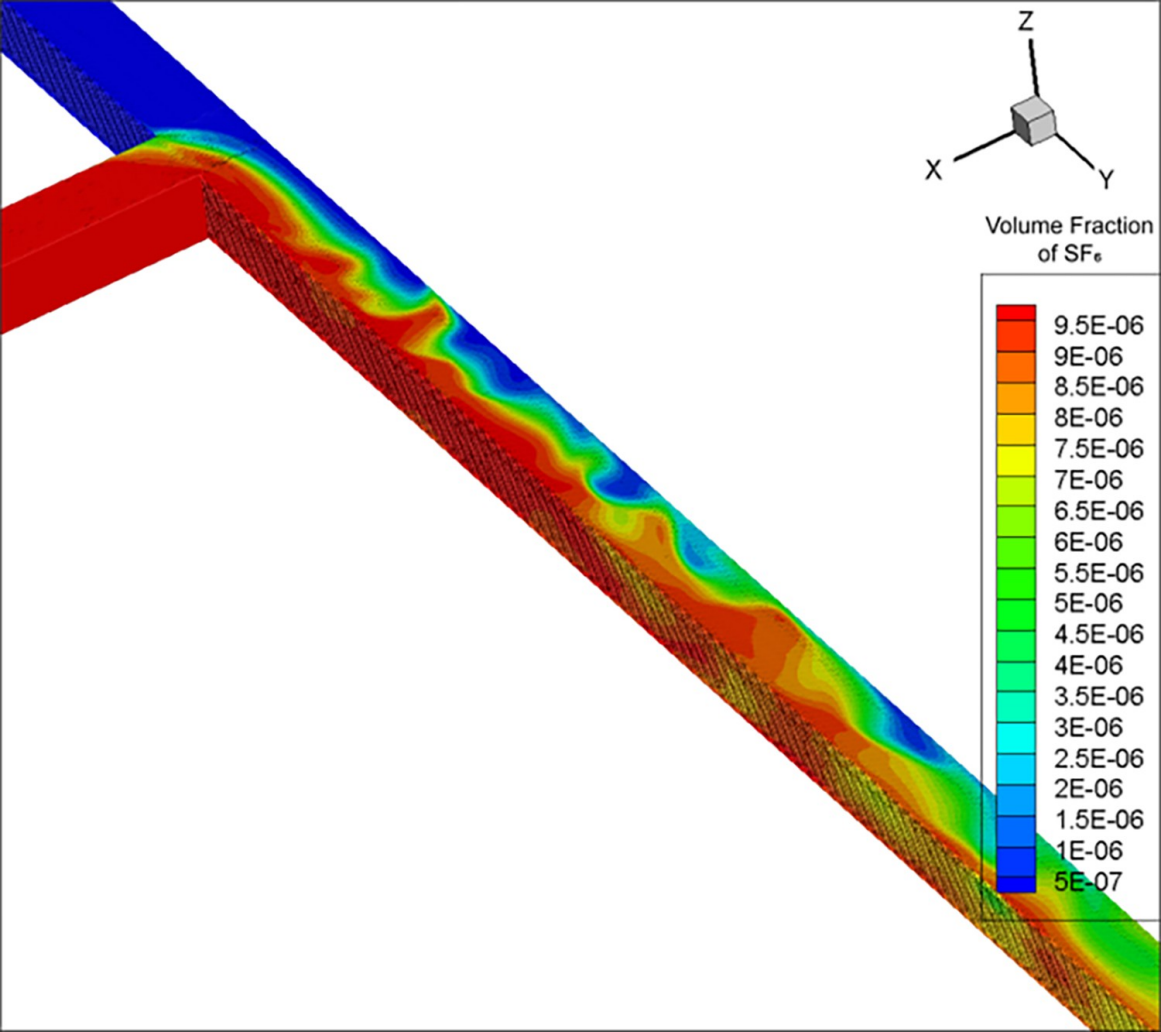
SF_6_ concentration distribution map calculated by the DES model.

After calculating the simulation parameters (Table 1), for the convenience of comparison with experimental results, the concentration data was kept accurate to 0.1 ppm. As a result, the mixing distance was calculated using the RNG k-ε model to be 32.8 m. Due to eddy current effects, the mixing distance calculated by the DES model fluctuated in the range of 27–30 m and was not stable; a value of 28.8 m was obtained once the calculation stopped at a certain timestep. The volume fractions in Figs 2–4 are dimensionless, and range from 0 to 1. cE−05 is c×10^−5^.

The SF_6_ concentration calculated by the (i) RNG k-ε model had a regularly layered distribution (Fig 3), and (ii) DES model showed an irregular wavy pattern (Fig 4). Under the influence of obstacles, turbulence, and flow velocity gradients, the fluid formed vortex-like flow states (vortices). Due to the DES model considering both turbulence and eddy currents, the effect of the latter caused SF_6_ to rotate alongside, forming an irregular distribution. The RNG k-ε model did not consider vortices due to turbulence, but only the natural and turbulence-related gas diffusion, resulting in a regular SF_6_ diffusion.

**Fig 5:**
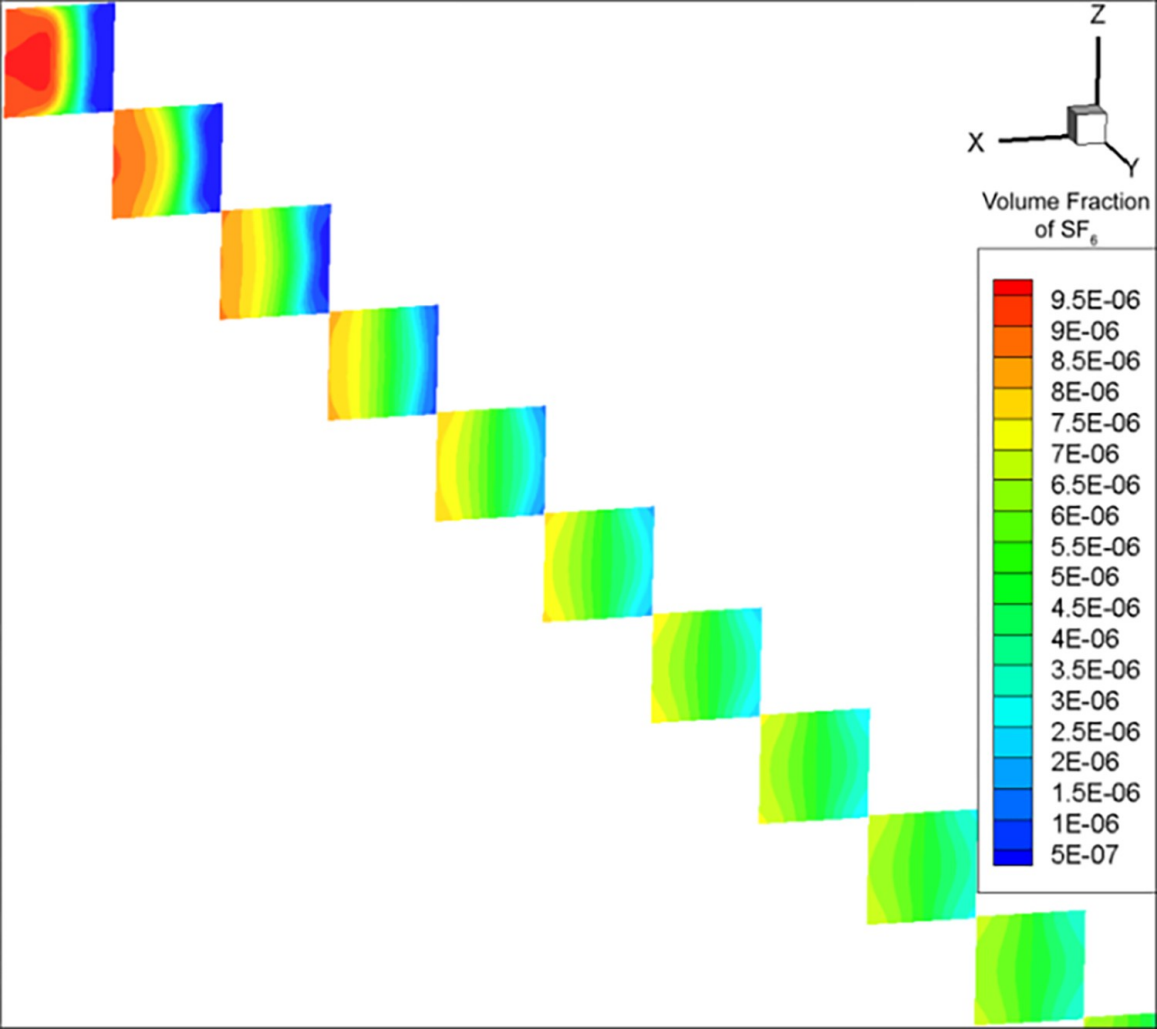
SF_6_ concentration distribution cross-sections taken every 1 m within the upstream of duct 3 in the RNG k-ε model.

**Fig 6:**
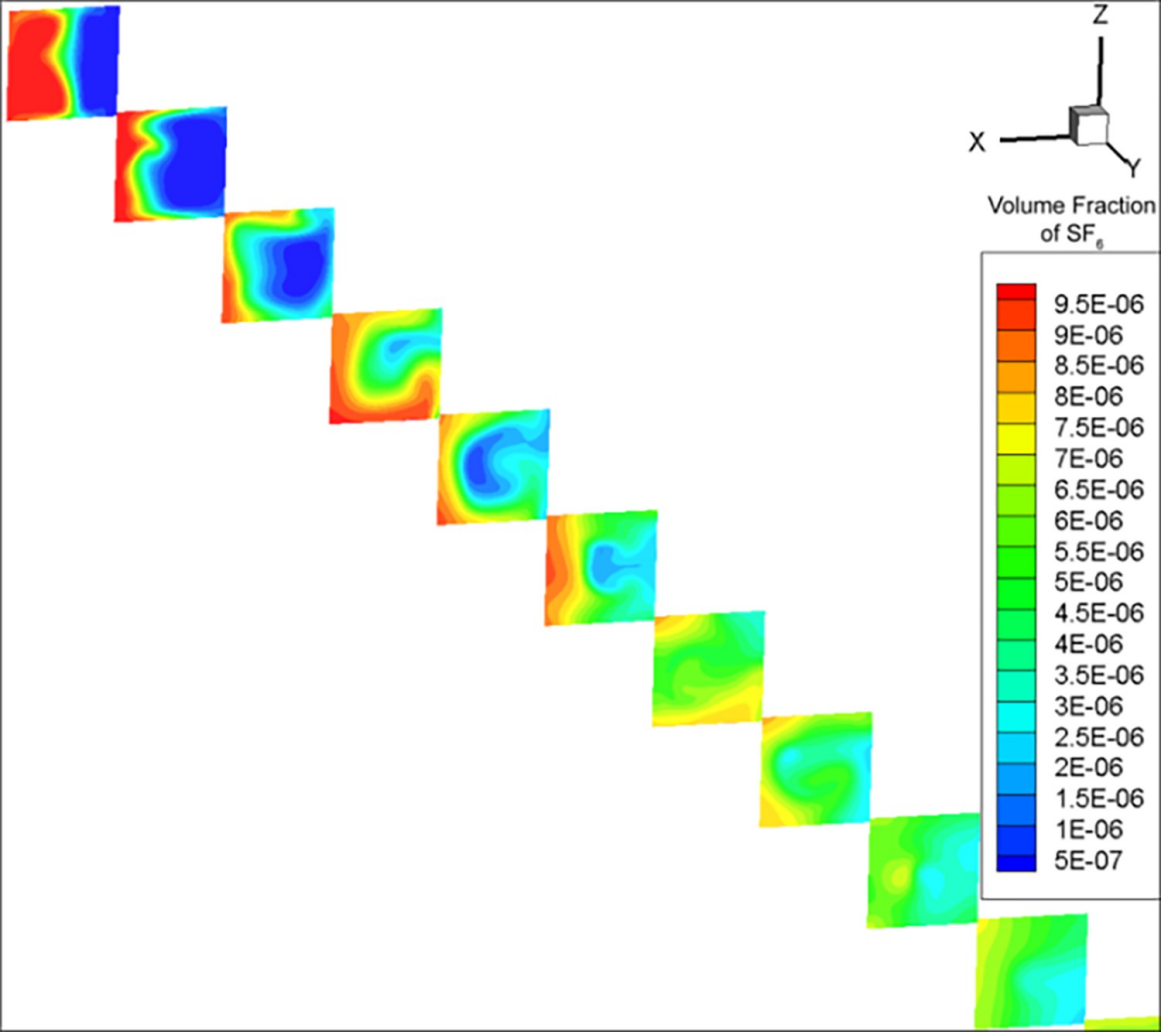
SF_6_ concentration distribution cross-sections taken every 1 m within the upstream of duct 3 in the DES model.

The equidistant SF_6_ concentration measurements in duct 3 calculated by the RNG k-ε model were relatively regular (Fig 5). The areas with higher SF_6_ concentrations were always distributed on the side with higher incoming SF_6_ concentration, with a concentration gradient in the negative *x*-axis direction. Under the DES model, SF_6_ exhibited a vortex-like pattern due to the influence of eddy currents (Fig 6). Although the areas with high SF_6_ concentrations at most stages were still roughly on the positive *x*-axis direction, the results of the DES model were more irregular and the positions of the maximum and minimum SF_6_ concentrations were not fixed.

**Fig 7:**
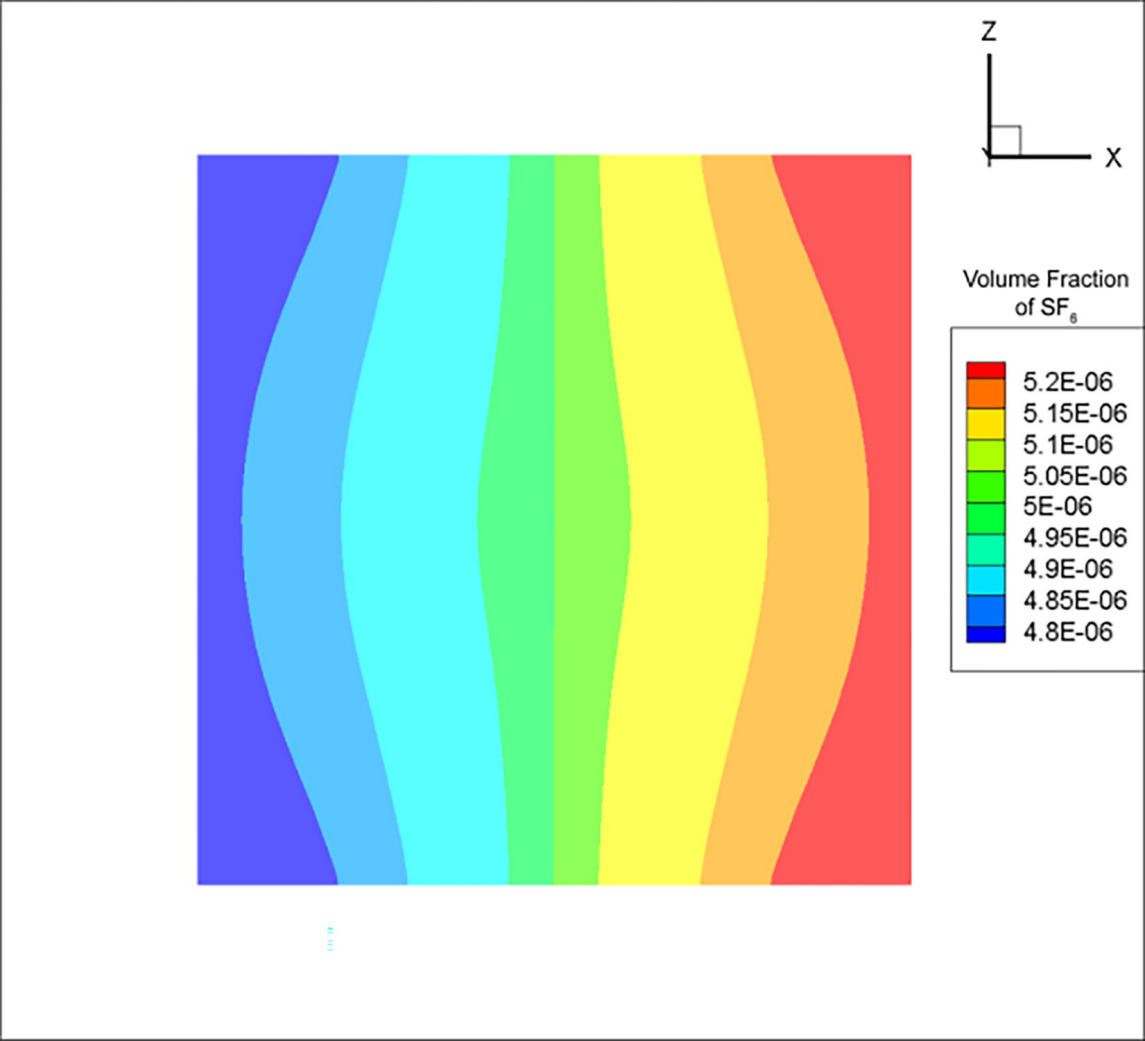
Cross-sectional SF_6_ concentration distribution at the uniform mixing distance in air duct 3 for the RNG k-ε model.

**Fig 8:**
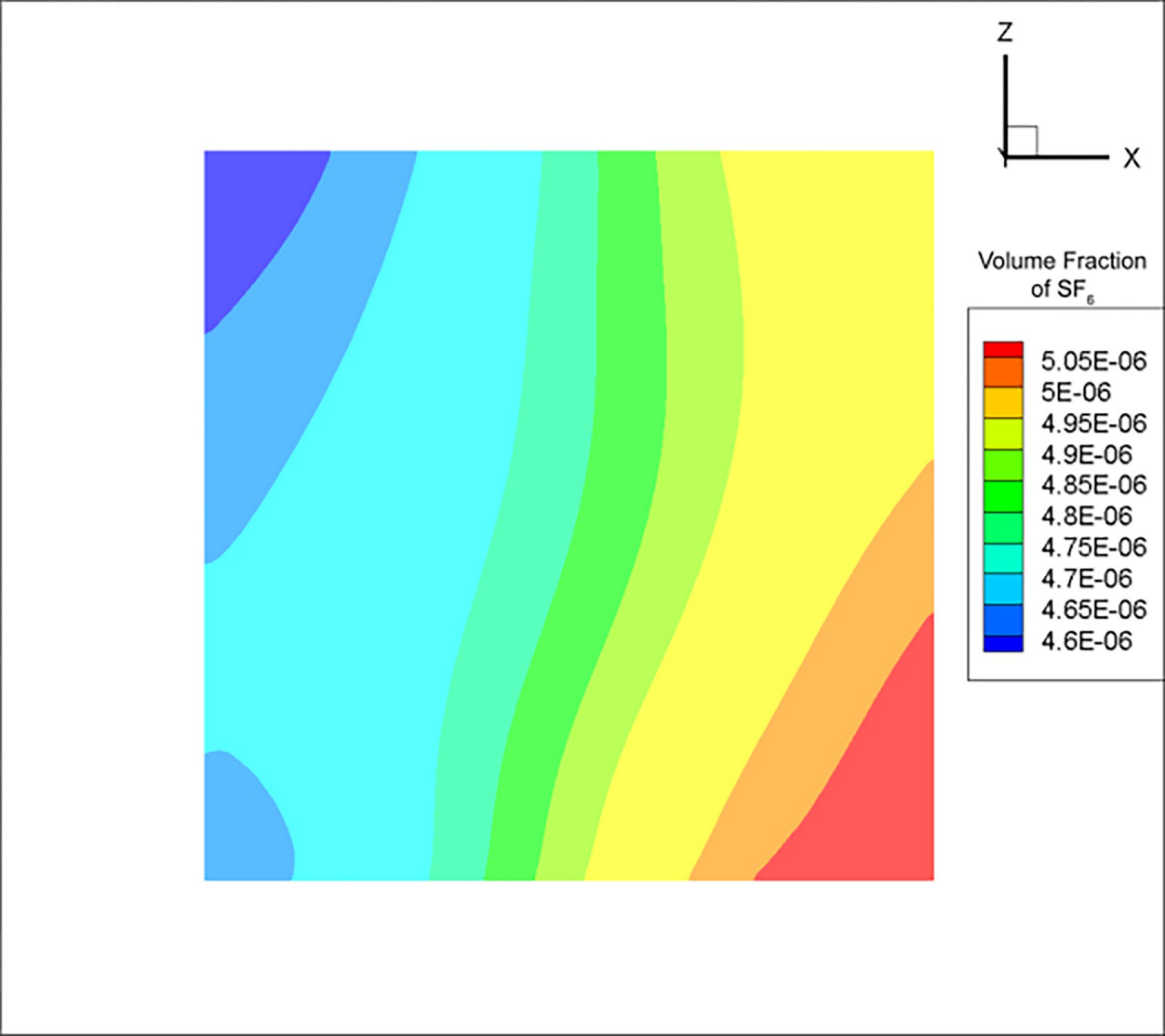
Cross-sectional SF_6_ concentration distribution at the uniform mixing distance in air duct 3 for the DES model.

Due to the cross-sectional area of duct 3 being much smaller than that of the mine tunnel, the influence of gravity on the small-scale pipeline tracer gas turbulence was minimal. Therefore, at the point of uniform mixing, the calculated SF_6_ concentration using the RNG k-ε model tended to have a regular left-right symmetric relationship with the region of high concentration (Fig 7). On the other hand, the DES model concentration distribution was relatively chaotic due to the effect of eddy currents, with the minimum and maximum concentrations located in the upper left and lower right corners, respectively (Fig 8).

## 3 Experimental and simulation results comparison

**Fig 9:**
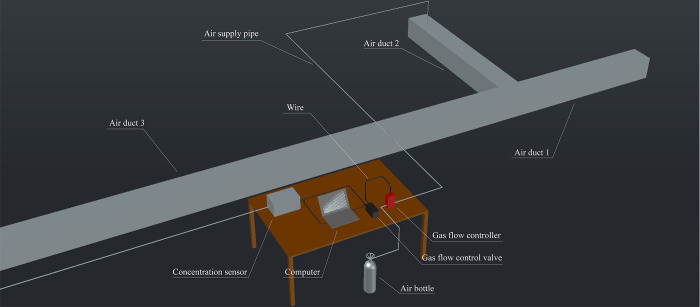
Schematic representation.

**Fig 10:**
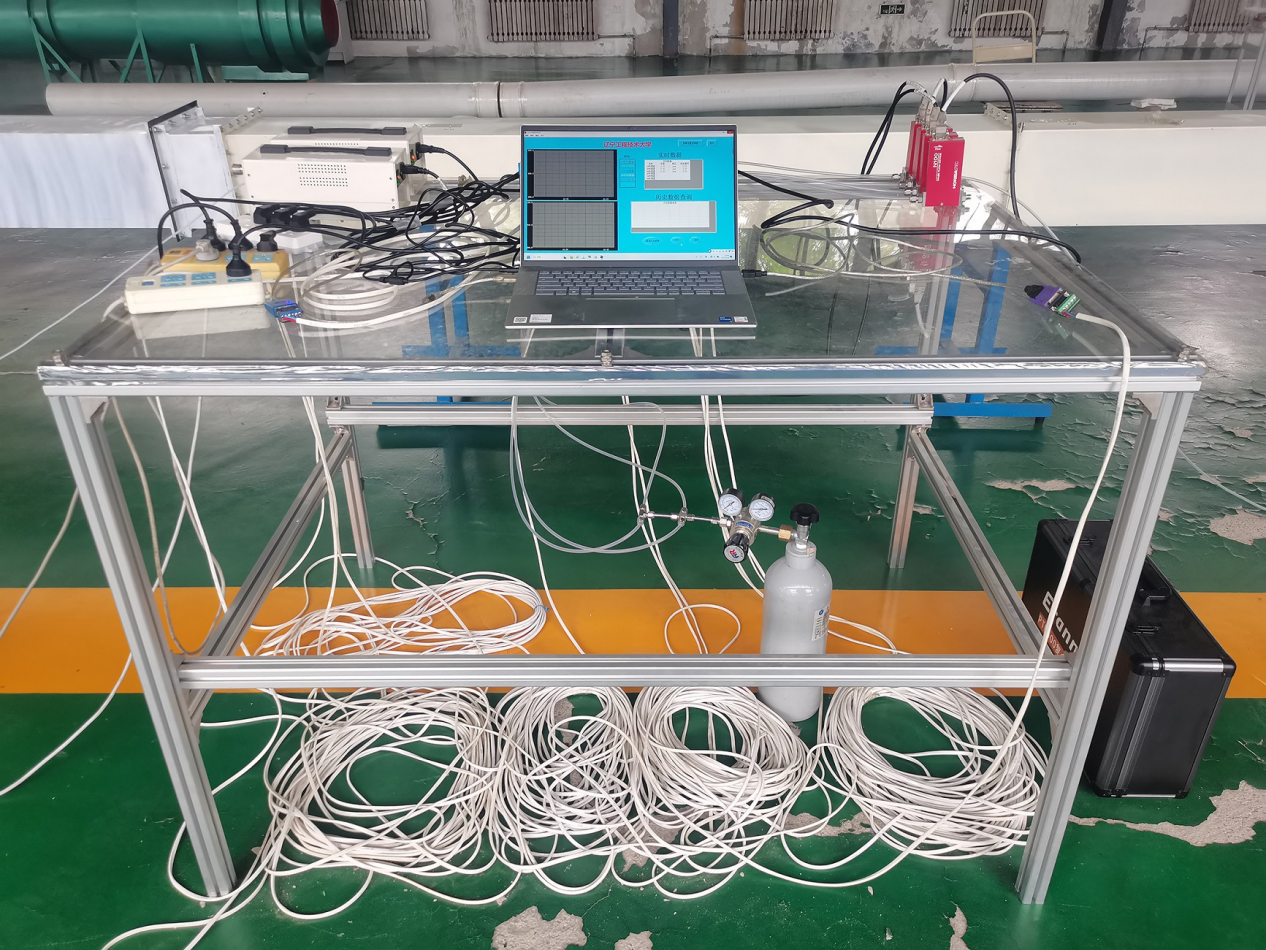
Photograph of the experimental setup.

The experimental setup (Figs 9 and 10) included a T-shaped cross air duct to simulate a mine tunnel, with two intake air ducts (1 and 2) measuring 3.0 m in length and a collection air duct 3 measuring 35.0 m in length. The air ducts were composed of several smaller ducts (each measuring 2.5 m in length), connected using sealing rubber rings and bolts. The SF_6_ gas flow controller was connected to the gas cylinder through a pipeline. The gas flow control valve was connected to the SF_6_ gas flow controller and a computer which input the flow data to control the gas release flow rate. The release port of the gas flow controller was connected to the air supply pipe, entering the intake duct with stirring fan blades to mix SF_6_ for an even distribution before flowing to the intersection point; this setup vastly increased the experiment accuracy. An SF_6_ concentration sensor was used to observe and record the concentration data with an accuracy of 0.1 ppm. The inlet of the sensor was connected to the gas supply pipe to sample the gas at the test position in the collection duct and measure the SF_6_ concentration. The air outlet of the collection duct was connected to the fan to provide wind power. The two intake ducts were connected to louvers. The wind speed adjustment range of duct 3 in this experimental system is 6-12m/s. The maximum release flow rate of the gas flow controller was 500 mL/min.

The relationship between the release flow rate, *Q*, of the gas flow controller and the average SF_6_ concentration in duct 2 can be expressed as

$$Q={60\mathrm{Sv}}_{2}\bar{c},$$ (9)

where S is the cross-sectional area of the air duct, m^2^; v_2_ is the wind speed in inlet duct 2, m/s; and $\bar{c}$ is the average SF_6_ concentration in duct 2, ppm.

### 3.1 Experimental process

Firstly, all equipment was switched on and the wind speed of the two intake air ducts, with a cross-sectional area of 0.09 m^2^, was adjusted to 3 m/s. For an SF_6_ concentration of 10 ppm in duct 2, the release flow rate was calculated to be 162 mL/min using Eq (9). After inspection, the overall leakage rate of the experimental air duct was found to be ~ 3%. To ensure a uniform SF_6_ concentration of 5 ppm in the collection air duct, the release flow rate was set to 168 mL/min.

We obtained SF_6_ concentration measurements at the midpoints of four edges, 4 corners, and the center of the cross-sections taken at 1-m intervals in duct 3. The concentration at a certain location was recorded only if the value displayed by the sensor remained stable for 10 s. We determined the maximum and minimum SF_6_ concentrations for each cross-section. After measurement, the airflow started at a distance of 16 m from the intake duct 3. The experimental measurement results are shown in Table 2.

**Table 2:** Maximum and minimum SF_6_ concentrations at the corners of cross-sections taken at 1-m intervals in duct 3.

Measurement points	Top	Below	Left	Right	Upper-left corner	Lower-left corner	Upper-right corner	Lower–right corner	Center
Concentration (ppm)									
Distance (m)									
1	0.5	0.4	0.0	9.5	0.0	0.0	11.4	8.5	4.3
2	0.4	4.1	0.5	7.4	0.3	1.4	6.7	9.0	5.4
3	5.0	4.8	2.3	6.9	2.3	2.2	7.9	6.2	4.7
4	5.1	7.4	2.9	7.7	3.6	2.5	6.9	7.5	5.6
5	5.2	6.4	3.5	6.3	3.1	3.9	7.1	5.9	4.9
6	4.5	5.7	4.0	6.6	3.6	4.6	6.2	6.9	5.2
7	5.1	5.3	4.0	6.0	4.8	4.2	5.5	6.9	4.6
8	4.5	5.2	4.2	5.8	3.8	4.4	5.5	5.9	5.1
9	4.9	5.1	4.0	5.8	4.5	3.9	5.5	6.1	4.8
10	4.6	5.3	4.3	5.5	4.1	4.4	5.2	5.8	5.2
11	4.6	5.1	4.1	5.6	3.9	4.3	5.4	6.0	4.9
12	4.2	4.9	3.9	5.5	3.3	4.1	5.2	5.9	4.5
13	5.0	3.9	4.1	4.4	4.2	3.9	5.7	4.1	4.4
14	4.9	4.9	4.5	5.1	4.3	4.6	5.6	5.1	4.8
15	5.0	4.8	4.6	5.4	4.4	4.6	5.6	4.9	5.1
16	4.7	5.2	4.6	5.4	4.4	4.9	5.2	5.6	4.9
17	4.9	5.2	4.5	5.5	4.3	4.6	5.4	5.7	5.0
18	4.9	5.4	4.7	5.6	4.3	5.0	5.5	5.6	5.1
19	4.7	5.1	4.5	5.4	4.4	4.7	5.1	5.6	4.8
20	4.9	5.2	4.7	5.4	4.5	5.0	5.2	5.4	5.3
21	5.0	5.1	4.7	5.2	4.5	4.6	5.3	5.5	5.0
22	4.9	5.3	4.8	5.3	4.5	5.0	5.2	5.6	5.1
23	4.9	5.0	4.5	5.4	4.5	4.6	5.3	5.5	4.9
24	5.0	4.9	4.7	5.4	4.6	4.6	5.3	5.4	4.9
25	5.0	5.1	4.8	5.3	4.6	4.9	5.2	5.3	5.0
26	4.9	5.2	4.7	5.2	4.7	4.8	5.2	5.3	5.1
27	5.0	5.2	4.8	5.3	4.6	4.9	5.3	5.4	5.1
28	5.0	5.1	4.8	5.3	4.7	4.9	5.3	5.3	5.0
29	4.9	5.0	4.7	5.3	4.7	4.7	5.2	5.3	5.0
30	4.9	5.1	4.7	5.2	4.7	4.8	5.2	5.3	4.9
31	5.0	5.0	4.9	5.1	4.8	4.8	5.1	5.2	5.0
32	5.0	5.0	4.8	5.2	4.8	4.8	5.2	5.2	5.0
33	5.0	5.0	4.8	5.2	4.8	4.8	5.2	5.2	5.0

### 3.2 Comparative analysis of experimental and simulation results

**Fig 11:**
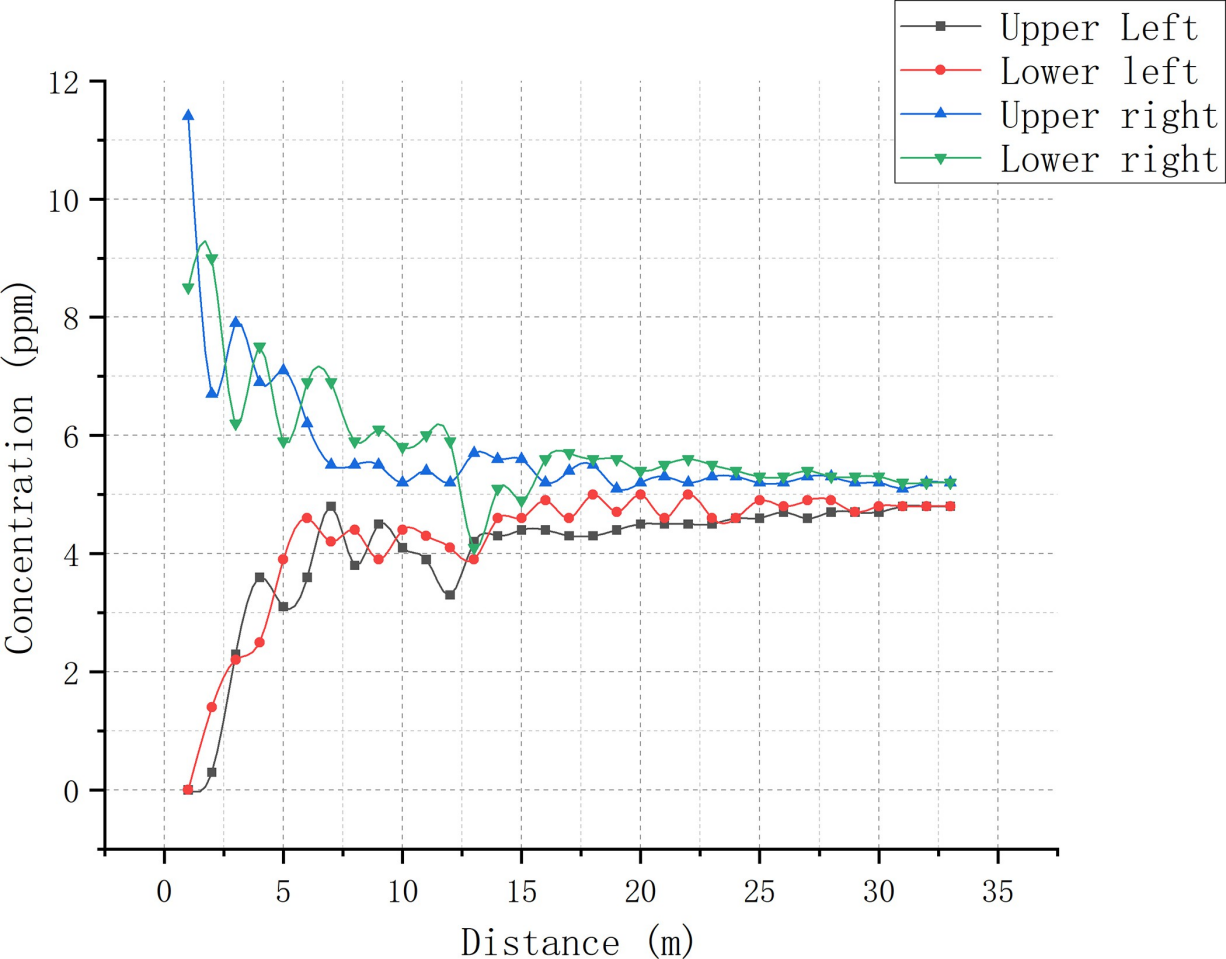
SF_6_ concentration curves at the four corners of each cross-section taken for duct 3.

As the minimum and maximum SF_6_ concentrations were almost stably distributed in the corners, only these values of each cross-section were recorded to determine the mixing distance. Draw a concentration curve graph (Fig 11).

The results of Table 2 and Fig 6 are shown. The vertical direction of the airflow in the two intake ducts resulted in clear vortex formation upon convergence, leading to an unstable SF_6_ concentration distribution at the duct 3 positions closer to the intersection. However, as the distance from the intersection point increased, the size of the vortex gradually decreased and the airflow stabilized. At and beyond 16.0 m, with the *y*-axis as the line of sight, the maximum and minimum SF_6_ concentrations of each cross-section were stably distributed in the lower- and upper-right corners, respectively. At 30.5 m, the minimum-to-maximum SF_6_ concentration ratio reached > 90%, achieving the standard of uniform mixing.

We set the simulated concentration precision to 0.1 ppm and compared it with the experimental results to analyze the accuracy of the RNG k-ε and DES models (Table 3).

**Table 3:** SF_6_ concentration comparison for the RNG k-ε and DES models with experimental data.

Distance (m)	Minimum SF_6_ concentration (ppm)			Maximum SF_6_ concentration (ppm)		
	RNG k-ε	DES	Experiment	RNG k-ε	DES	Experiment
1	0.0	0.0	0.0	9.9	10.0	11.4
2	0.0	0.0	0.3	9.7	10.0	9.0
3	0.0	0.2	2.2	9.5	9.5	7.9
4	0.3	1.8	2.5	8.3	9.6	7.7
5	0.9	0.5	3.1	8.7	9.3	7.1
6	1.3	1.5	3.6	8.3	9.2	6.9
7	1.6	3.3	4.2	7.9	8.3	6.9
8	2.0	2.3	3.8	7.6	8.2	5.9
9	2.3	2.6	3.9	7.4	6.8	6.1
10	2.6	2.4	4.1	7.1	7.3	5.8
11	2.8	3.1	4.1	7.0	6.8	6.0
12	3.0	3.7	4.1	6.8	6.1	5.9
13	3.2	3.9	4.2	6.6	5.4	5.7
14	3.4	4.4	4.3	6.5	6.0	5.6
15	3.5	4.4	4.4	6.4	6.4	5.6
16	3.6	3.8	4.4	6.3	6.0	5.6
17	3.8	4.1	4.3	6.2	5.3	5.7
18	3.9	4.7	4.4	6.0	5.7	5.6
19	4.0	4.8	4.4	6.0	5.7	5.6
20	4.0	4.6	4.5	5.9	5.5	5.4
21	4.2	3.9	4.5	5.8	5.5	5.5
22	4.2	4.1	4.5	5.8	5.0	5.6
23	4.3	4.5	4.5	5.7	5.3	5.5
24	4.4	4.4	4.6	5.6	5.1	5.4
25	4.4	4.6	4.6	5.6	5.3	5.3
26	4.5	4.6	4.7	5.5	5.4	5.3
27	4.5	4.5	4.6	5.5	5.1	5.4
28	4.6	4.5	4.7	5.4	5.2	5.3
29	4.6	4.5	4.7	5.4	4.9	5.3
30	4.6	4.5	4.7	5.4	4.7	5.3
31	4.6	4.7	4.8	5.3	4.9	5.2
32	4.7	4.5	4.8	5.3	4.7	5.2
33	4.7	4.4	4.8	5.2	4.7	5.2

**Fig 12:**
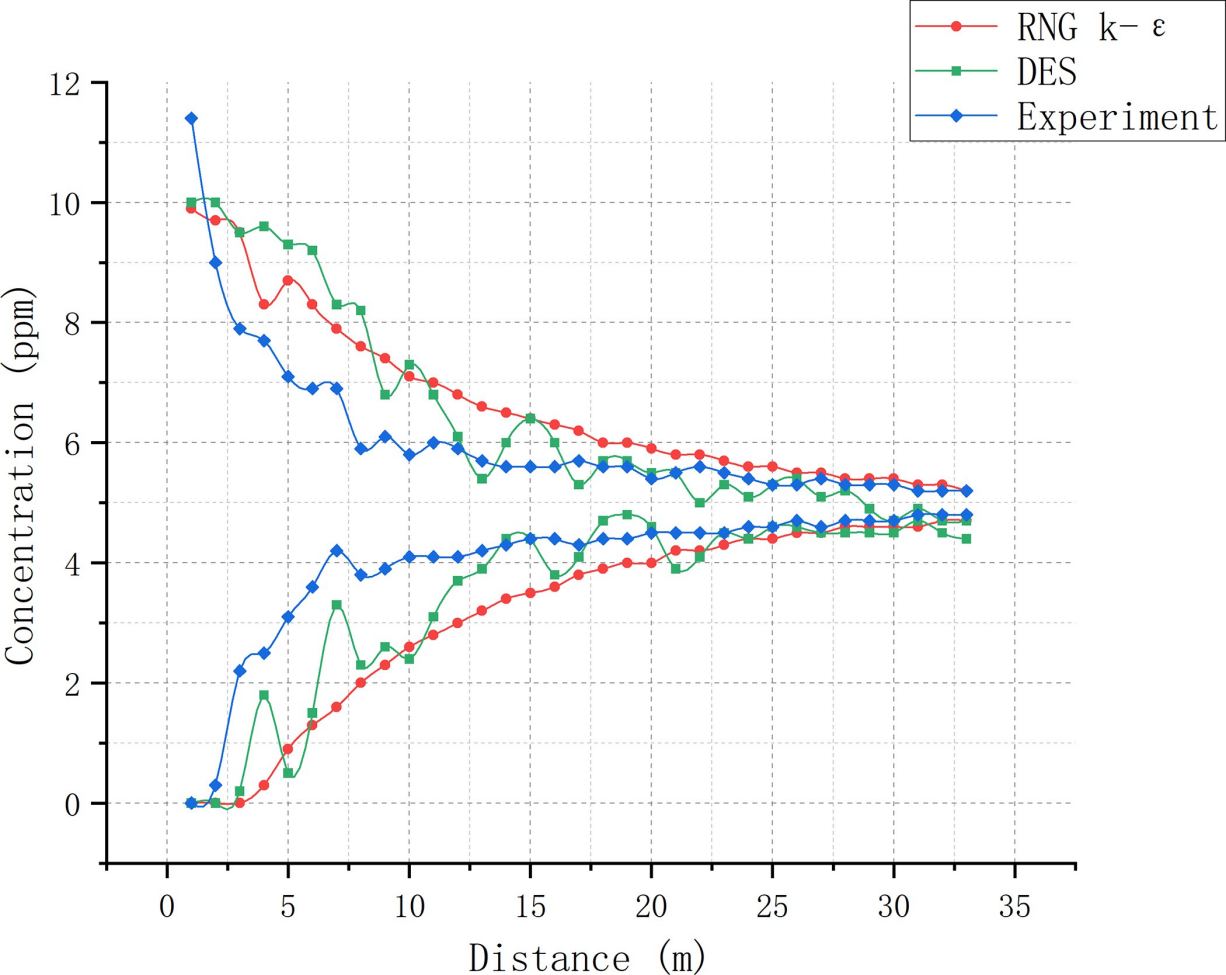
SF_6_ concentration curve comparison between simulations and experimental data from various cross-sections within duct 3.

**Fig 13:**
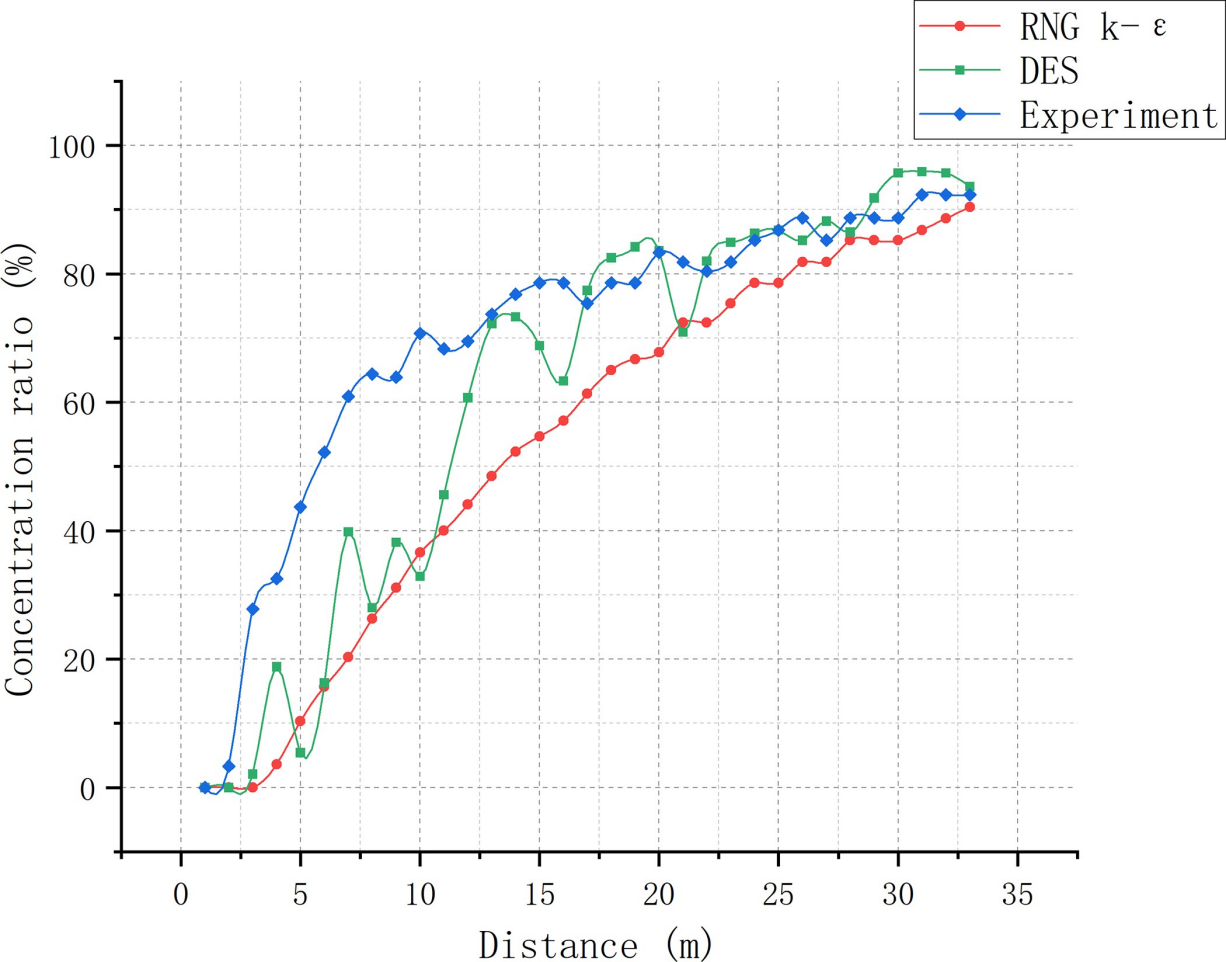
Minimum-to-maximum SF_6_ concentration ratio curve comparison between simulations and experiment.

In the 1–7 m range of duct 3, the minimum-to-maximum SF6 concentration ratio in the experiment changed significantly faster than in the simulations, indicating that the SF_6_ mixing rate in the experiment was much faster (Figs 12 and 13; Table 3). However, as the concentration ratio crossed 60% of its maximum value at the 7-m mark, the mixing rate reduced in comparison to the simulations due to the weakening of eddy currents. After the 26-m mark, the concentration ratio between simulated and experimental data tended to be consistent, both crossing 80%. The mixing distances for the RNG k-ε model, DES model, and experiment were 32.8, 28.8, and 30.5 m, respectively, with a < 10% error rate for the two models compared to the experimental results. Due to the inevitable presence of slight air leakage in the setup, the external air entering from the air duct gap also facilitates gas mixing. Therefore, the ideal mixing distance is speculated to be slightly longer than our experimental data, i.e., slightly closer to that of the RNG k-ε model. In the real environment, the SF_6_ concentration distribution is influenced by both large- and small-sized eddies. Smaller eddies can make the mixing process more stable and rapid. However, as the DES model neglects eddies smaller than the grid size, its SF_6_ concentration is relatively unstable, with a strongly fluctuating concentration ratio. The average SF_6_ concentration at the uniform mixing point calculated by the DES model is 4.85 ppm, which has the largest error compared to the experimental results. The RNG k-ε model computed an average SF_6_ concentration of 4.95 ppm at the point of uniform mixing. Under the influence of large-sized eddies, the concentration distribution and average concentration fluctuations throughout the SF_6_ flow were more pronounced in this case. However, the presence of small-sized eddies in this experiment assisted gas mixing and reduced the SF_6_ concentration fluctuation amplitude compared to the DES model. The RNG k-ε model ignored all vortices, resulting in the smallest concentration ratio fluctuation amplitude.

### 3.3 Error analysis of simulation and experimental results

**Fig 14:**
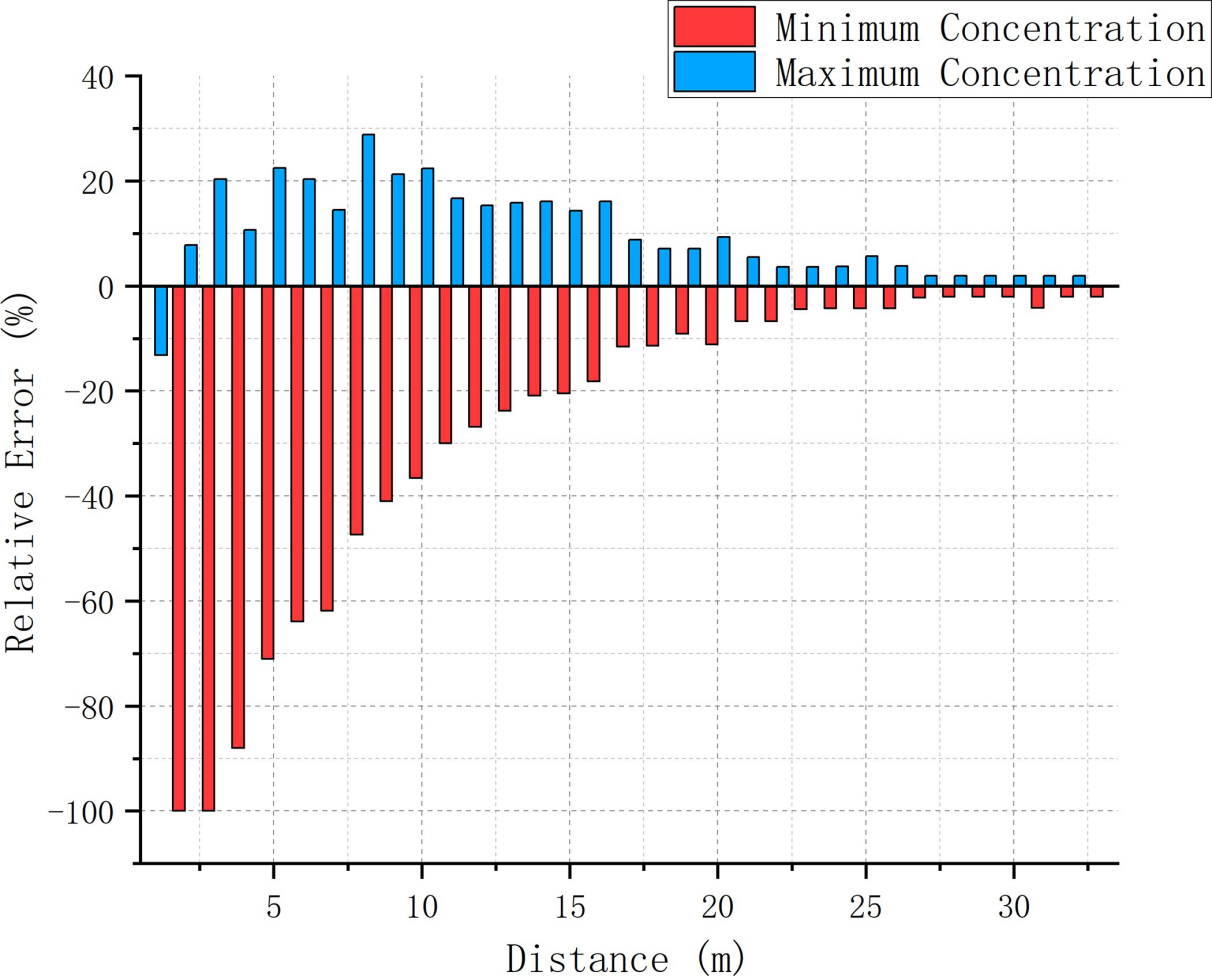
Relative error between the experimental results and the RNG k-ε model results.

**Fig 15:**
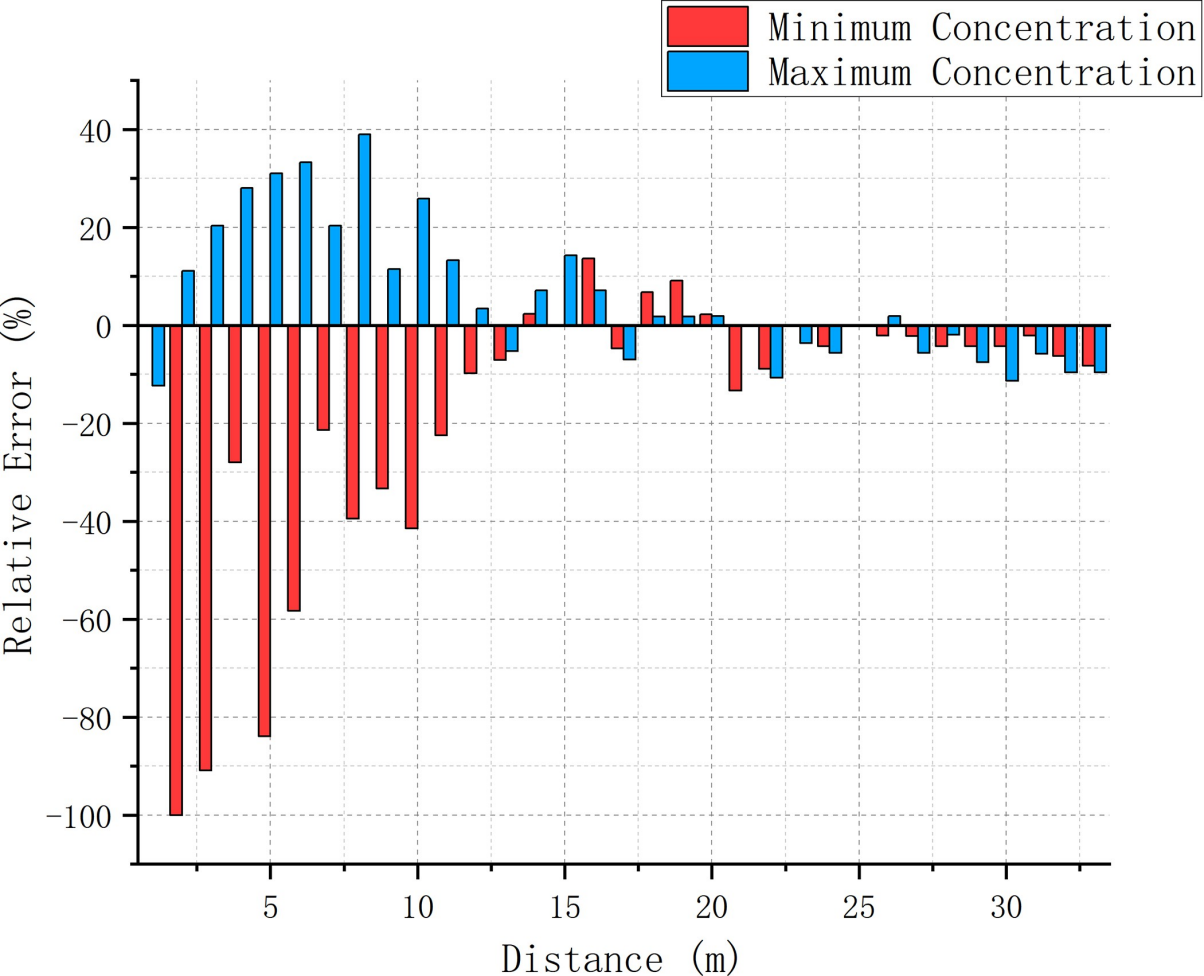
Relative error between the experimental results and the DES model results.

We analyzed the relative errors between the two numerical simulation models and the minimum and maximum concentrations at various positions in the experiment (Figs 14 and 15). We observed that as the distance increased, the errors of both models decreased. The error variation between the RNG k-ε model and experimental results was regular, stable, and decreasing with increasing distance; the minimum (maximum) concentration error was always negative (positive) above 1 m. The error variation between the DES model and the experimental results was, however, quite chaotic. This difference was observed due to the relatively stable concentration variations in the RNG k-ε model and experimental results compared to the large fluctuations in the DES model due to ignoring the instability caused by small-scale eddies. Up to a distance of 20 m, the large eddy current effect led to a small error between the DES model and experimental results, beyond which it destabilized and led to strong fluctuations. No decreasing pattern was observed in this case, and the error rate occasionally exceeded 10%. However, the error between the RNG k-ε model and experimental results maintained stability for all distances and gradually decreased (the error of the minimum and maximum concentrations remained within 10%). At this stage, the overall error of the DES model was greater than that of the RNG k-ε model; comprehensive comparison revealed the latter to be superior in determining the mixing distance.

In the experiment, under different wind speeds and SF_6_ release rates, the mixing distance always fluctuated in the range of 29.5–30.5 m. Therefore, we confirmed that in a small-sized air duct with completely turbulent airflow, the mixing distance is minimally affected by the wind speed and SF_6_ release rate.

## 4 Comparison of mixing distances between air ducts and real mine tunnels

There exists a roadway with a rectangular cross-section and a characteristic length of about 4 meters in reality [30]. Based on the actual size of the mine tunnel, we established a cross tunnel model with a cross-sectional area of 4 m × 4 m with the same shape as the air duct model in Section 2. The dimensions of the tunnel and air duct models were proportional to the grid, and the tunnels were labeled 1, 2, and 3 in the corresponding order. The wind speed for tunnels 1 and 2 was set to 2 m/s; the SF_6_ concentration in tunnel 2 was set to 80 ppm; and the wall roughness was set to 0.05 m; other parameter settings remained the same as in Table 1. According to the Coal Mine Safety Regulations [31], the wind speed in tunnels should be within the exact range of 0.25–8.00 m/s. To ensure this, along with 30 ≤ y+ ≤ 300 for tunnel 3, the first grid height of the tunnel model was set to 0.012. Using the RNG k-ε method, the SF_6_ mixing distance in the tunnel was computed to be 335 m.

The analytical solution for the three-dimensional turbulent diffusion at point (x, z) of the cross-section at y during the continuous and constant release of tracer gas can be expressed as [10, 32]

$$c=\frac{m}{4\pi E_{r}y}exp(-\frac{(x^{2}+z^{2})v}{16E_{r}y}),$$ (10)

$$E_{r}=0.032\sqrt{\alpha }{\mathrm{LvRe}}^{-0.04},$$ (11)

$$\alpha =\frac{\lambda \rho }{8},$$ (12)

where E_r_ is the lateral diffusion coefficient, m^2^/s; m is the mass of the gas released per unit time, kg/s; x and z are the horizontal and vertical coordinates in the inner diameter direction of duct 3, respectively, m; and y is the longitudinal coordinate within duct 3, m; α is the friction resistance coefficient of the tunnel or air duct, kg/m^3^.

Based on the work of Nikuradse et al. [12], in a fully turbulent state, we have

$$\lambda =\frac{1}{{\left({2\log}_{10}\frac{3.7L}{\Delta }\right)}^{2}},$$ (13)

where λ is the friction coefficient of the tunnel or air duct; and Δ is the absolute roughness of the tunnel or air duct, m.

According to Eq (12), the similarity relationship between the mixing distance and the size of the air duct and tunnel, as well as the roughness of the wall, can be derived as

$$\frac{y_{1}}{y_{2}}={\left(\frac{L_{1}}{L_{2}}\right)}^{1.04}\log_{\frac{{3.7L}_{2}}{\Delta_{2}}}\frac{{3.7L}_{1}}{\Delta_{1}}.$$ (14)

In the experimental air duct, we fixed the mixing distance to y_1_ = 30.5 m, the characteristic length to L_1_ = 0.3 m, and the roughness to Δ_1_ = 0.0005 m. The characteristic length and roughness of the tunnel were L_2_ = 4 m and Δ_2_ = 0.05 m, respectively. The calculated mixing distance of the tunnel was 333.1 m, which was consistent with the RNG k- ε simulation result of 335.0 m. For intake tunnel wind speeds of 1.0, 1.5, 2.0, 2.5, 3.0, 3.5, and 4.0 m/s, the mixing distances were 350.0, 343.0, 335.0, 330.0, 327.0, 325.0, and 324.0 m, respectively, with errors within 5%.

## 5 Conclusion

This study compared and analyzed the SF6 concentration data and mixing distance calculated and experimentally measured by RNG and DES models. The results showed that in a 0.3×0.3m cross duct, the blending distances calculated by RNG k-ε model and DES model were 32.8m and 28.8m, respectively. The blending distance in the experiment was 30.5m, which was basically consistent. And the mixing distance remained almost unchanged under different wind speeds and SF6 release amounts in the experiment. The RNG k-ε model revealed the longest mixing distance, while demonstrating a higher accuracy and stability. It also offers a low computational overhead. Therefore, the RNG model is deemed far superior to the DES model. Although both models are unable to accurately calculate the SF_6_ concentration distribution before uniform gas mixing, in practical application, only the mixing distance needs to be computed. Concentration sensors can be installed at or beyond the mixing distance from the inlet of the tunnel to accurately measure the airflow. Therefore, the RNG k-ε model already meets the needs of practical wind measurements. After similarity verification, the mixing distance computed for the experimental model and the RNG k-ε mine tunnel model essentially conforms to the similarity relationship, indicating that the RNG k-ε numerical simulations can accurately calculate the mixing distance. For the practical installation of SF_6_ concentration sensors for tunnel wind measurement in the future, the proposed numerical simulation method can be used to determine the approximate sensor placement positions, making the process time-efficient.

SF_6_ has several advantages as a tracer gas. However, as it is also a greenhouse gas, in practical applications, it is necessary to maintain a low release amount. The average SF_6_ concentration range in a tunnel during the release process must be maintained in the range of 5–100 ppm, ensuring accurate measurement, while minimizing environmental impact. Future works must attempt to discover a tracer gas that does not adversely affect the environment and has a density closer to air in order to employ the proposed numerical simulation method to determine the mixing distance.
